# Tumor-associated Endo180 requires stromal-derived LOX to promote metastatic prostate cancer cell migration on human ECM surfaces

**DOI:** 10.1007/s10585-015-9765-7

**Published:** 2015-11-13

**Authors:** Matthew P. Caley, Helen King, Neel Shah, Kai Wang, Mercedes Rodriguez-Teja, Julian H. Gronau, Jonathan Waxman, Justin Sturge

**Affiliations:** Department of Surgery & Cancer, Imperial College London, London, W12 0NN UK; Centre for Cutaneous Research, Blizard Institute, Barts & The London School of Medicine & Dentistry, Queen Mary University of London, London, E1 2AT UK; Division of Cancer Studies, King’s College London, New Hunts House, Guys Campus, London, SE1 1UL UK; Human Oncology and Pathogenesis Program, Memorial Sloan-Kettering Cancer Center, New York, NY 10065 USA; Louis V. Gerstner, Jr. Graduate School of Biomedical Sciences, Memorial Sloan-Kettering Cancer Center, New York, NY 10065 USA; School of Biological, Biomedical & Environmental Sciences, University of Hull, Cottingham Road, Hull, HU6 7RX UK; Departamento de Genética, Facultad de Medicina, Universidad de la República (UDELAR), Montevideo, Uruguay

**Keywords:** Bone, Cell migration, Collagen, Fibroblast, Osteoblast, Prostate cancer

## Abstract

The diverse composition and structure of extracellular matrix (ECM) interfaces encountered by tumor cells at secondary tissue sites can influence metastatic progression. Extensive in vitro and in vivo data has confirmed that metastasizing tumor cells can adopt different migratory modes in response to their microenvironment. Here we present a model that uses human stromal cell-derived matrices to demonstrate that plasticity in tumor cell movement is controlled by the tumor-associated collagen receptor Endo180 (CD280, CLEC13E, KIAA0709, MRC2, TEM9, uPARAP) and the crosslinking of collagen fibers by stromal-derived lysyl oxidase (LOX). Human osteoblast-derived and fibroblast-derived ECM supported a rounded ‘amoeboid-like’ mode of cell migration and enhanced Endo180 expression in three prostate cancer cell lines (PC3, VCaP, DU145). Genetic silencing of Endo180 reverted PC3 cells from their rounded mode of migration towards a bipolar ‘mesenchymal-like’ mode of migration and blocked their translocation on human fibroblast-derived and osteoblast-derived matrices. The concomitant decrease in PC3 cell migration and increase in Endo180 expression induced by stromal LOX inhibition indicates that the Endo180-dependent rounded mode of prostate cancer cell migration requires ECM crosslinking. In conclusion, this study introduces a realistic in vitro model for the study of metastatic prostate cancer cell plasticity and pinpoints the cooperation between tumor-associated Endo180 and the stiff microenvironment imposed by stromal-derived LOX as a potential target for limiting metastatic progression in prostate cancer.

**Electronic supplementary material:**

The online version of this article (doi:10.1007/s10585-015-9765-7) contains supplementary material, which is available to authorized users.

## Introduction

Metastatic bone disease (MBD) affects approximately 1 million advanced cancer patients per annum in the EU, USA and Japan; and estimates suggest that approximately one fifth of MBD cases result from advanced prostate cancer [1]. MBD is normally accompanied by the presence of additional metastatic lesions in visceral organs. However, in vitro experimental systems used to study putative metastatic targets tend to overlook the precise composition, organization and bioactivity of human bone and visceral tissues. The de novo extracellular matrix (ECM) produced by human trabecular bone osteoblasts is abundant in the minerals, proteins and growth factors found in normal human bone, which provides an accurate biomaterial to study therapeutic targets in the context of MBD [2–4]. Likewise, human fibroblast-derived ECM has been used to develop more realistic in vitro models of human cancer localized in visceral tissue in which its influence on therapeutic strategies can be considered [5, 6].

Tumor cells can adopt different modes of migration during metastasis. Three modes of tumor cell migration include grouped, bipolar and rounded, which respectively involve: (a) collective ‘epithelioid-like’ cell clusters directed by a leader cell; (b) ‘mesenchymal-like’ translocation of single cells coordinated by forward protrusion and rear retraction of the plasma membrane; and (c) ‘amoeboid-like’ forward translocation of singular spheroidal cells [7, 8]. Tumor cells can switch back-and-forth between different modes of migration in response to external and/or internal cues. This type of morphological plasticity is a feature of the epithelial-to-mesenchymal, mesenchymal-to-amoeboid and collective-to-amoeboid transitions that occur during tumor progression [9, 10]. The ‘amoeboid-like’ cell phenotype predominates at the invasive edge of high-grade tumors [11] and has been identified as an escape mechanism from some anti-invasive strategies [12]. Tumor cells engaged in this rounded mode of cell migration do not require focal adhesion turnover as they do for bipolar ‘mesenchymal-like’ migration [13]. Instead rounded cell migration is driven by the spatial localization of integrins and cytoskeletal regulators at the posterior plasma membrane [14, 15] and generation of RhoA and Rho kinase associated protein kinase (ROCK)-based actinomyosin contractile signals [16].

The type I transmembrane collagen receptor Endo180 (CD280, CLEC13E, KIAA0709, MRC2, TEM9, uPARAP) is as a strong prognostic indicator for prostate cancer survival [17, 18]. Within this context Endo180 functions as a modulatory switch for epithelial-to-mesenchymal transition (EMT), and pro-invasive behavior in normal prostate epithelial cells triggered by increased crosslinking and stiffness of the basement membrane following its exposure to advanced glycation end-products (AGEs) [17, 18]. The pro-migratory and pro-invasive role of Endo180 involving the promotion of RhoA-ROCK-based actinomyosin contractility at the cell posterior [17–19] has been confirmed in a range of tumor and stromal cell types, both in vivo and in vitro, using ectopic over expression, genetic silencing, genetic ablation or targeted blockade of receptor function [17–30]. Given the expression of Endo180 observed in tumor cell foci in metastatic bone lesions [4], and increased levels of soluble Endo180 in the serum of patients with visceral and bone metastases [31], we hypothesized that Endo180 can regulate prostate cancer cell plasticity on the bone-like ECM derived from human osteoblasts and visceral tissue-like ECM derived from human fibroblasts.

Lysyl oxidase (LOX) is a copper-dependent amine oxidase that is produced by osteoblasts and fibroblasts to give tissue its structural support and mechanical stiffness by crosslinking the adjacent collagen fibers that they deposit as part of the ECM [32, 33]. LOX plays a fundamental role in metastasis [34–37], including the formation of the pre-metastatic lesions in bone that are colonized by circulating tumor cells and expand into occult osteolytic metastases [38]. Considering the positive cooperation between tumor-associated Endo180 and AGE-dependent crosslinking and stiffness of basement membrane matrix [18], we hypothesized that Endo180-dependent prostate cancer cell migration cooperates with LOX-dependent crosslinking of the ECM derived from human osteoblasts and fibroblasts.

## Materials and methods

### Cells and cell culture

For osteoblast isolation approximately fifty post-operative human trabecular bone chips of 1–2 mm^2^ were washed thoroughly in PBS to remove hematopoietic cells and incubated for 2 h at 37 °C in 10 ml of 1.2 mg/ml type IV collagenase diluted in DMEM (Invitrogen Ltd. Paisley, UK). Supernatants containing digested cellular components (>10^7^ cells) were harvested and cultured at 37 °C in 5 % CO_2_ in a 1:1 mix of DMEM and F-12 medium (Invitrogen Ltd.) supplemented with 10 % v/v FBS (First Link UK Ltd., Birmingham, UK), 2 mM l-glutamine, 100 U/ml penicillin, 100 mg/ml streptomycin and 0.25 mg/ml amphotericin B (Invitrogen Ltd.). Primary human osteoblasts and HCA2-hTERT human fibroblasts were maintained in DMEM +10 % v/v FCS, 1 mM penicillin/streptomycin and 2 mM l-glutamine.

PC3, DU145 and VCAP cells were maintained in RPMI medium (Invitrogen Ltd.) +10 % v/v FBS, 1 mM penicillin/streptomycin and 2 mM l-glutamine. Endo180 knockdown and control cells were generated by transfection (Qiagen Superfect) of PC3 cells with the shRNA vector pRNATin-H1.2/Hygro containing coral GFP (Antibodies-Online GmbH, Aachen, Germany) and shEndo180 or non-targeting shEndo180 scrambled control (shSCN) sequence inserts [26, 39] that were evaluated previously to rule out any off-target effects on other pro-migratory and pro-invasive proteins [17, 19]. Transfected cells were selected in fully supplemented DMEM containing hygromycin-B (20 µg/ml) (Santa Cruz Biotechnology Inc., Heidelberg, Germany, UK). PC3 cells were transfected with the pcDNA3-Endo180 or empty pcDNA3 vector using lipofectamine and selected with G418 (0.5 mg/ml), as previously described [26, 39].

### Matrix preparation and analysis

Rat type I collagen derived from rat tails was commercially sourced (#354236, BD Biosciences, Oxford, UK) and used at a concentration of 50 µg/ml in 0.02 M glacial acetic acid to coat wells following the manufacturers instructions. For human cell-derived ECM production HCA2 fibroblasts and primary human trabecular bone osteoblasts were seeded at a density of 1.5 × 10^4^ cells per well in 96-well plates (#CLS3595, Corning^**®**^ Costar^**®**^). Confluent cultures of HCA2 fibroblasts were stimulated with 2 mM ascorbate to induce the production of native ECM. Confluent cultures of primary human trabecular bone osteoblasts were stimulated with 100 µM ascorbate, 10 nM dexamethasone and 10 mM β-glycerophosphate to induce mineralized ECM production as previously described [2, 4]. Stimulation media were replaced every 2 days. After 10 days cells were washed with PBS (3 × 5 min) and removed by three successive freeze–thaw cycles (in PBS) and incubation in 1 % w/v sodium deoxycholate for 5 min. Resulting decellularized matrices were washed with PBS (5 × 5 min) before use. Mineralization was determined by von Kossa staining (5 % w/v silver nitrate), as previously described [4].

BAPN (0.1–1.0 mM) was included in stimulation media of fibroblasts and osteoblasts during the 10-day period of matrix generation to inhibit LOX-dependent type I collagen fiber crosslinking. After 10 days the matrices generated in the presence of BAPN were decellularized. The inhibitory effect of BAPN on collagen crosslinking in fibroblast-derived ECM and osteoblast-derived ECM was ascertained by adapting a previously described method [37]. In brief, decellularized ECM derived from fibroblasts and osteoblasts (untreated or treated with BAPN) were immunostained with rabbit anti-human type I collagen polyclonal antibody (R1038X, Acris Antibodies GmbH, Herford, Germany) and secondary anti-rabbit Alexa Fluor 488-conjugated IgG (Invitrogen Ltd.). Images of the type I collagen fibers present in fibroblast-derived ECM and osteoblast-derived ECM were acquired using Image Xpress^MICRO^ (IXM) (Molecular Devices UK Ltd., Wokingham UK) [4]. The curvature ratio (defined as *x/y*, where *x* = the total length of each fiber and *y* = the linear distance between the start and end of each fiber) was calculated using Image J software (arbitrary units) in images of type I collagen fibers. The same images were processed using Metamorph® software (Molecular Devices UK Ltd.) to calculate integrated fluorescent signal intensity per unit area (µm^2^) using a modification of a protocol used to calculate the integrated fluorescent signal intensity of type I collagen fibers per cell [4].

### Cell morphology and migration assays

Cells were seeded at a density of 2 × 10^3^ cells per well on plastic, type I collagen, fibroblast-derived ECM and osteoblast-derived ECM in 96-well Optilux Black/Clear Bottom plates (#734-0395, VWR International Ltd., Lutterworth, UK) and allowed to adhere prior to image acquisition at a rate of 1 frame every 30 min for 24 h using IXM set to live cell imaging mode (5 % v/v CO_2_ at 37 °C). Segmented images were used to manually score the number of cells that were undergoing ‘grouped’ versus ‘singular’ cell migration at time zero (0 h) and at each 6-h time point of for the 24-h experimental time frame. In the case of ‘singular’ cell migration cells were scored according to a bipolar versus rounded mode of cell migration. The predominant mode of cell migration was classified according to the criteria presented in Table 1. The % of cells with each phenotype was then used to calculate the rounded/bipolar ratio at time zero (0 h) and at each 6-h time point of the 24-h experimental time frame. The average cell velocities (μm/h) were calculated from the cell trajectories generated from manual cell tracking and post analysis (Metamorph® software). For all conditions tested the tracks from ≥100 cells were used to calculate the average cell velocities.

**Table 1:** Criteria used for scoring different modes of prostate cancer cell migration

Migratory mode	Cellular morphology observed during translocation	Criteria used to define the predominant mode of migration
Grouped	Epithelioid; as part of a moving cell cluster or participation in frequent interactions with adjacent cells	>80 % cells display epithelioid morphology during translocation
Singular, bipolar	Mesenchymal; movement as elongated singular cell with defined leading edge and retraction of trailing uropod	Rounded/bipolar ratio <1.0
Singular, rounded	Amoeboid; movement as spheroid without a retracting uropod	Rounded/bipolar ratio >1.0
Singular, mixed	Equal numbers of cells with mesenchymal and amoeboid morphologies	Rounded/bipolar ratio = 1.0

### Flow cytometry

Cells were trypsinized and fixed in 4 % w/v paraformaldehyde (10 min), blocked and permeabilized in immunofluorescence buffer (IFB: 4 % w/v BSA and 1 % v/v FBS) containing 0.2 % w/v saponin. Cells were pelleted and incubated with anti-human Endo180 primary monoclonal antibody (A5/158, E1/183 or 39.10) diluted in IFB (1 h), washed in IFB (3 × 5 min), incubated with Alexa Fluor-555 conjugated secondary antibody diluted in IFB (1 h) and washed IFB (3 × 5 min). Cells were pelleted, resuspended in PBS and assessed by flow cytometry (BD FACS Canto, BD, Oxford, UK). Gating was performed with unstained cells and cells stained with isotype matched IgG.

### Immunoblot analysis

Protein concentrations in whole cell lysates were determined using a Pierce BCA protein assay kit. Equal amounts of protein were resolved by SDS-PAGE using 7 % w/v polyacrylamide gels and electroblotted onto PVDF membranes, which were incubated at room temperature in blocking buffer (PBS + 5 % w/v BSA) (1 h) then primary antibody (anti-Endo180 A5/158 mAb; anti-GAPDH) diluted in blocking buffer at 4 °C (16 h). After washes in PBS+ 0.1 % v/v Tween^®^-20 (PBS-T) (5 × 5 min) blots were incubated in HRP-conjugated goat anti-mouse or goat anti-rabbit IgG diluted in blocking buffer (1 h). Blots were washed in PBS-T (5 × 5 min) prior to visualization of immunoreactive bands using chemiluminescence.

### Cell adhesion assay

Cell adhesion could not be measured using crystal violet because the fibroblast-derived and osteoblast-derived ECM retained the stain. Instead, cells were seeded at a density of 1.5 × 10^4^ per well of a 96-well plate onto test substrata and incubated for 1 h before washing in PBS and addition of culture medium containing CellTiter-Glo^®^ buffer (Promega, UK) at a ratio of 1:1 and final volume of 200 µl. Plates were mixed vigorously for 2 min to induce cell lysis and the contents of each well transferred to opaque 96-well plates (Corning^®^ Costar^®^; Z37 185-8; Sigma Aldrich Ltd., Poole, UK). Luminescence was measured on a PHERAstarPlus plate reader (BMG LabTech, Aylesbury, UK).

### MTT cell proliferation assay

1.2 × 10^4^ cells per well were seeded onto test substrata in 24-well plates and incubated for 48 h. 5 mg/ml MTT was added and cells incubated for 3.5 h at 37 °C. Media was removed and 300 µl of extraction buffer (0.5 M dimethylformamide; 20 % w/v SDS) added per well followed by incubation for 2 h. 100 µl of buffer was transferred per well to a 96-well plate. Absorbance (570 nm) was measured using a Sunrise plate reader (Labtech International Ltd., Ringmer, UK).

### Statistical analysis

Student’s *t* test was performed using SPSS 15.0 software; p < 0.05 was considered significant.

## Results

### Generation of human stromal cell-derived ECM surfaces with LOX-dependent cross links

**Fig. 1:**
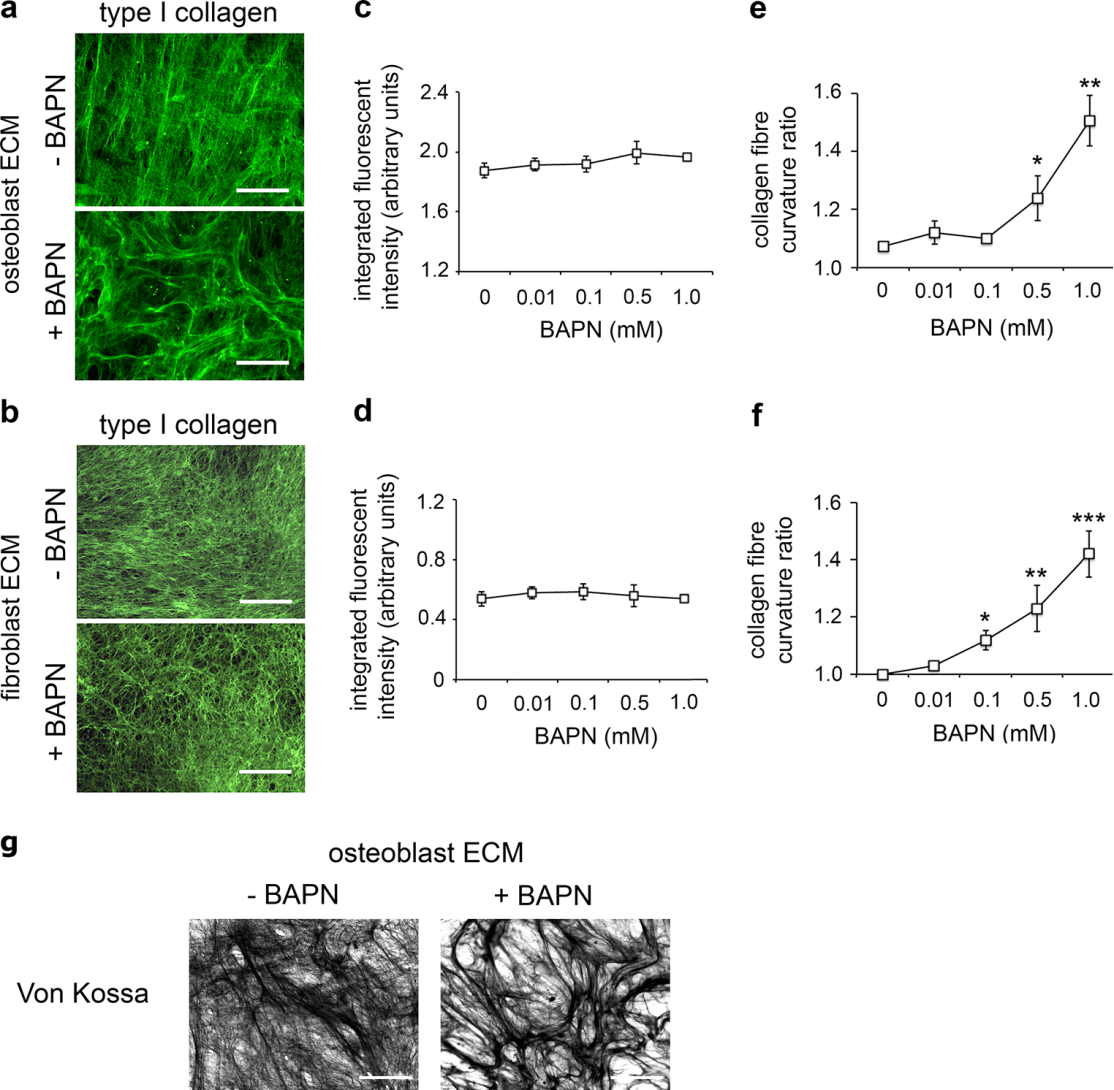
LOX inhibition increases type I collagen fiber curvature in matrices produced by human stromal cells. **a**, **b** Immunofluorescent images of type I collagen fibers produced by primary human trabecular bone osteoblasts (**a**) and human HCA2 dermal fibroblasts (**b**) in the absence (-BAPN) or presence (+BAPN) of LOX inhibitor (1 mM), *scale bar* = 50 μm. **c**, **d** Relative levels of total type I collagen deposited by osteoblasts (**c**) and fibroblasts (**d**) in the absence (–) or presence of BAPN (0.01–1 mM) (average ± s.d. of integrated fluorescent intensity of immunostaining per µm^2^). **e**, **f** Collagen fiber curvature ratio in the matrices deposited by osteoblasts (**e**) and fibroblasts (**f**) in the absence (–) or presence of BAPN (0.01–1 mM); 20 collagen fibers measured for each condition in duplicate. **e**, **f** Average ± s.d. values for three individual experiments, within which each experimental condition in duplicate, are shown; significant differences compared to control (no BAPN treatment) are indicated (*p < 0.05, **p < 0.001 and ***p < 0.0003). **g** Von Kossa staining of osteoblast derived ECM generated in the absence (−BAPN) or presence (+BAPN) of LOX inhibitor (1 mM), *scale bar* = 50 μm

ECM generation was induced in confluent monolayers of primary human trabecular bone-derived osteoblasts isolated from post-operative human trabecular bone and human HCA2 dermal fibroblasts. After 10 days immunofluorescent staining of type I collagen fibers was performed on decellularized osteoblast-derived ECM (Fig. 1a) and decellularized fibroblast-derived ECM (Fig. 1b). Image analysis revealed that inhibition of collagen crosslinking with the LOX inhibitor BAPN did not affect the total levels of type I collagen deposition by human osteoblasts (Fig. 1c) or HCA2 fibroblasts (Fig. 1d) but induced a significant increase in the curvature ratio of the collagen fibers in both types of matrices (Fig. 1e, f). Von Kossa staining of osteoblast-derived ECM indicated that BAPN treatment does not affect mineralization (Fig. 1g). These results confirm that human osteoblast-derived ECM and human fibroblast-derived ECM both require LOX to maintain their organized structure, thus providing two physiologically relevant substrata for studying how tumor-associated Endo180 modulates the plasticity of prostate cancer cell migration in the presence and absence of LOX-dependent collagen crosslinking.

### Rounded metastatic prostate cancer cell migration is favored on human bone matrix

**Fig. 2:**
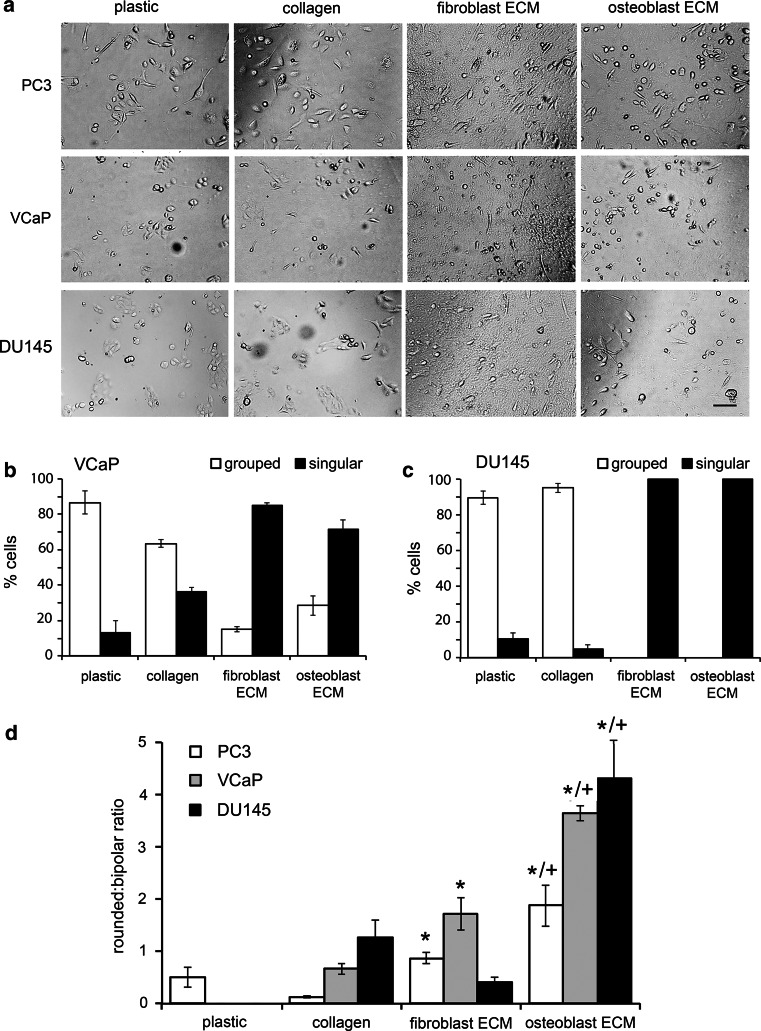
Human stromal cell-derived surfaces promote plasticity of movement in metastatic prostate cancer cells. **a** Stills extracted from Videos 1–12 in Online Resources 1–12 of PC3, VCaP and DU145 migrating on tissue culture plastic, commercial rat-tail type I collagen and native ECM generated by human HCA2 dermal fibroblasts (fibroblast ECM) and primary human trabecular bone osteoblasts (osteoblast ECM); *scale bar* = 50 μm. **b**, **c** The percentages of VCaP cells (**b**) and DU145 cells (**c**) participating in grouped and singular modes of cell migration on the four substrata. **d** The rounded:bipolar ratios for singular PC3, VCaP and DU145 cells migrating on the four substrata; significant increases compared to type I collagen (*p < 0.01) and fibroblast ECM (^+^p < 0.01) are indicated. **b**–**d** Average ± s.d. values for three individual experiments, within which each experimental condition was tested in quadruplicate, are shown

Three prostate cancer cell lines were included in the study: PC3 and VCaP that originate from bone metastatic lesions located in the lumbar vertebra [40, 41] and DU145 that originate from a soft tissue metastatic lesion in the parieto-occipital fissure of the cerebrum [42]. Time-lapse video microscopy was used to compare their migratory behavior on standard tissue culture plastic, commercial type I collagen, fibroblast-derived ECM and osteoblast-derived ECM, as seen in the videos (Online Resources 1–12) and corresponding video stills (Fig. 2a). All movies were scrutinized and the observed mode of migration for each cell in the field of view was scored according to the criteria presented in Table 1. In the case of the two epithelioid-like cell lines, VCaP and DU145, the proportion of cells engaged in grouped and singular modes of cell migration were calculated (Fig. 2b, c). Under all other experimental conditions >20 % of cells adopted a singular migratory mode, which was considered sufficient for the rounded/bipolar ratio of cell migratory mode to be calculated (Fig. 2d).

**Fig. 3:**
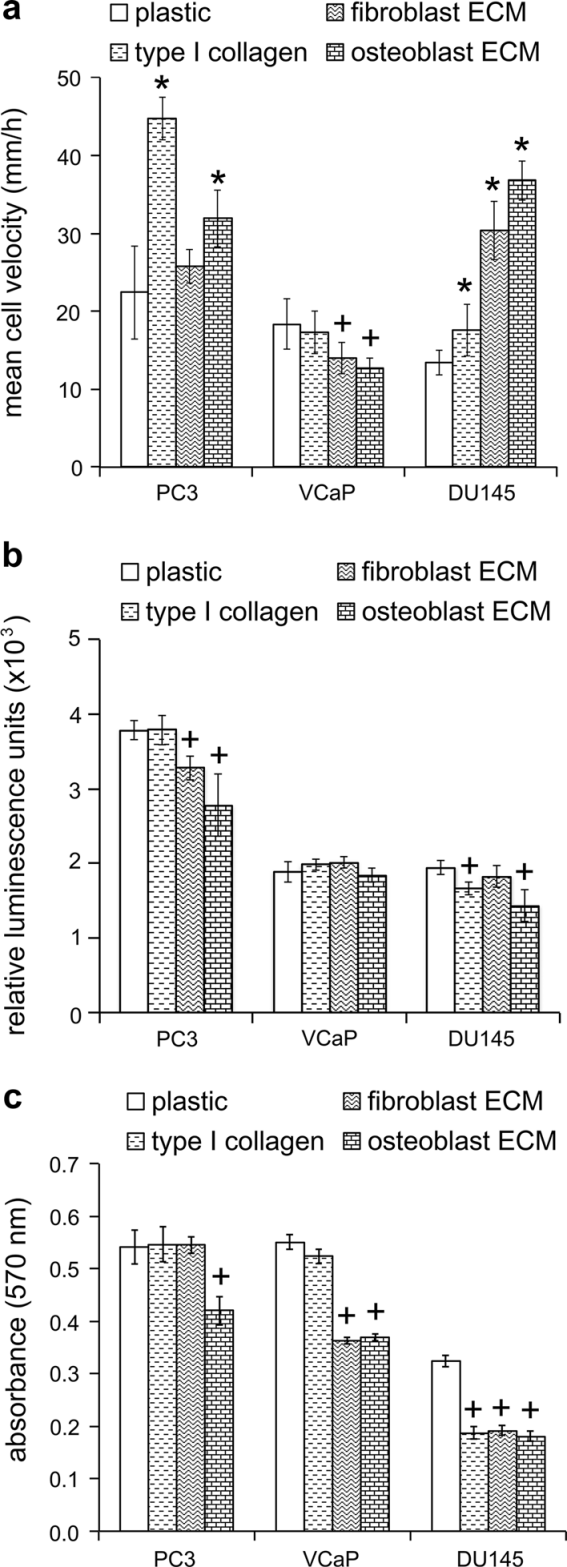
The migration, adhesion and proliferation rates of metastatic prostate cancer cells are differentially modulated on human stromal cell-derived ECM surfaces. **a**–**c** The migratory velocities (μm/h) over a 24 h time-frame (**a**), adhesion levels at 1 h (**b**) proliferation rates at 48 h (**c**) of PC3, VCaP and DU145 cells cultured on tissue culture plastic (plastic), rat-tail type I collagen (type I collagen), human HCA2 fibroblast-derived ECM (fibroblast ECM) and human trabecular bone osteoblast-derived ECM (osteoblast ECM). **a**–**c** Average ± s.d. values for three individual experiments, within which each experimental condition was tested in quadruplicate, are shown. Significant increases (*p < 0.05) and decreases (^+^p < 0.05) compared to plastic are indicated

PC3 and VCaP cells, but not DU145 cells, transitioned towards singular and bipolar modes of migration on collagen in comparison to tissue culture plastic (Fig. 2b–d). On the other hand, fibroblast-derived ECM supported singular mixed bipolar/rounded modes of migration for PC3 cells, a singular rounded mode of migration for VCaP cells and a singular bipolar mode migration for DU145 cells (Fig. 2d). Moreover, for all three cell-types the predominant mode of migration on osteoblast-derived ECM was singular and rounded (Fig. 2d). The rounded migration of PC3 and DU145 cells on osteoblast-derived ECM was associated with a high velocity (Fig. 3a), a decreased adhesion (Fig. 3b) and decreased proliferation (Fig. 3c) when compared to the same parameters on plastic. In contrast, the rounded migratory mode of VCaP cells that predominated on both fibroblast-derived and osteoblast-derived ECM was associated with a low velocity (Fig. 3a), no change in adhesion (Fig. 3b) and decreased proliferation (Fig. 3c) when compared to plastic. These data confirm that metastatic prostate cancer cells display plasticity in their mode of migration on human stromal cell-derived ECM.

### Endo180 is upregulated in metastatic prostate cancer cells in contact with human ECM

**Fig. 4:**
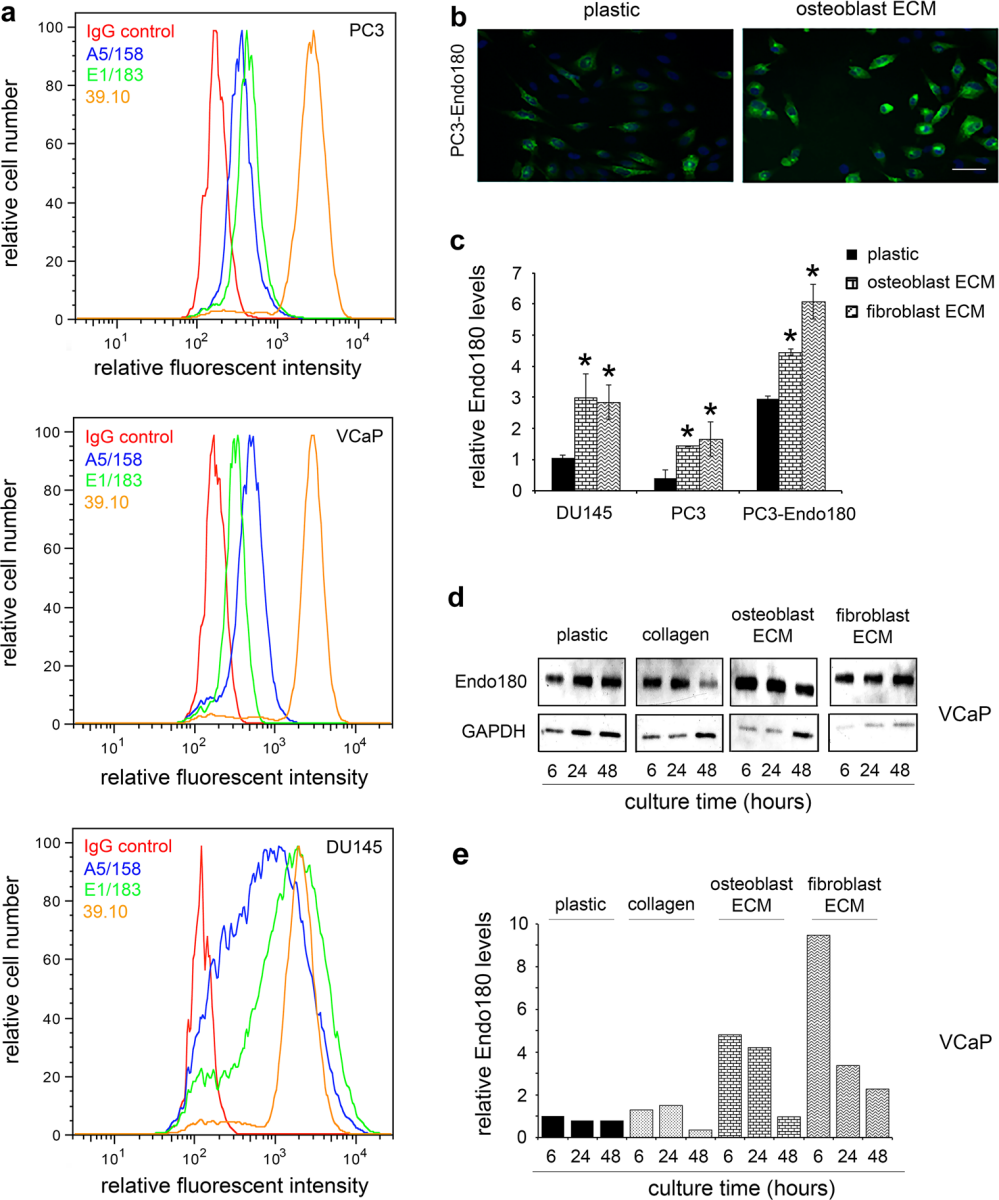
Endo180 is upregulated in metastatic prostate cancer cells cultured on human stromal cell-derived ECM surfaces. **a** Flow cytometry analysis of PC3, VCaP and DU145 cells using three anti-human Endo180 monoclonal antibodies (A5/158, E1/183, 39.10). **b** Immunofluorescent staining of Endo180 (A5/158 mAb) in PC3-Endo180 cells cultured on plastic and ECM generated by primary human trabecular bone osteoblasts (osteoblast ECM); *scale bar* = 50 μm. **c** Relative levels of Endo180 expression (integrated fluorescent intensity; A5/158 mAb immunostaining) in PC3, PC3-Endo180 and DU145 cells cultured on tissue culture plastic (plastic) and ECM generated by primary human trabecular bone osteoblasts (osteoblast ECM) and human HCA2 dermal fibroblasts (fibroblast ECM); average ± s.d. values for three individual experiments, within which each experimental condition was tested in quadruplicate, are shown; significant differences compared to plastic are indicated (*p < 0.05). **d** Immunoblot shows Endo180 expression (A5/158 mAb) in VCaP cells cultured on plastic, rat-tail type I collagen (collagen), osteoblast ECM and fibroblast ECM for 6, 24 and 48 h (GAPDH = loading control). **e** Relative levels of Endo180 expression in (**d**) quantified using densitometry (n = 1)

Flow cytometry analysis using three anti-human Endo180 monoclonal antibodies (A5/158, E1/183, 39.10) confirmed that the receptor is expressed in PC3, VCaP and DU145 cells (Fig. 4a). The differential Endo180 staining profiles of the three antibodies may reflect differences in their epitope engagement and/or masking by ligand binding and/or the open-closed conformational state of the receptor, as a molecular mechanism postulated in our two recent studies [17, 18]. Immunofluorescent staining analysis confirmed that Endo180 levels were significantly increased in DU145 cells, parental PC3 cells and PC3 cells that overexpress Endo180 (PC3-Endo180) cultured on human osteoblast-derived ECM and fibroblast-derived ECM (Fig. 4b, c). Endo180 appeared to cluster in ‘hot-spots’ in cells with a rounded phenotype (Fig. 4b). Immunoblot analysis and densitometry confirmed that Endo180 was also upregulated in VCaP cells cultured for 6-24 h on human osteoblast-derived ECM (up to 4-fold) and fibroblast-derived ECM (up to 9-fold) (Fig. 4d, e); immunoblotting results were similar for PC3 and DU145 cells (Supplementary Data Figure S1). The decrease in Endo180 levels at 48 h could be due to the depletion of factors that promote Endo180 expression in the bone microenvironment, such as transforming growth factor-beta-1 (TGFβ_1_). These findings are in accordance with results obtained using co-cultures of human trabecular bone-derived osteoblasts and DU145 or PC3 cells [4], Endo180 immunostaining of tumor cell foci in MBD [4] and its raised levels in the serum of patients with osseous and/or visceral metastases [31].

### Endo180 is required for rounded prostate cancer cell migration on human stromal ECM surfaces

**Fig. 5:**
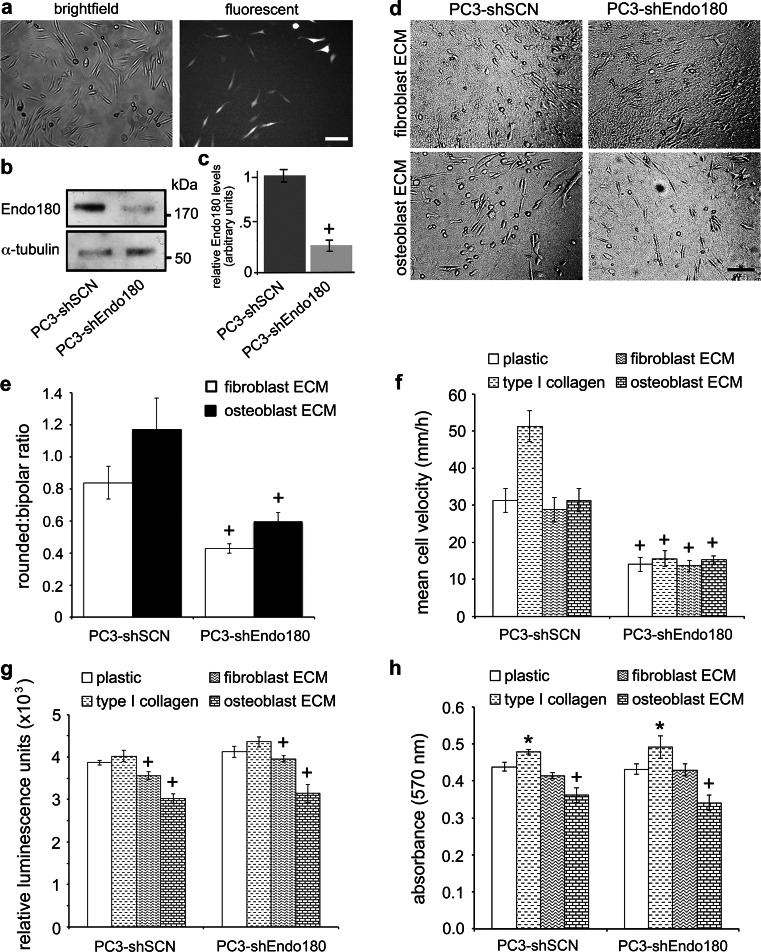
Silencing Endo180 inhibits metastatic prostate cancer cell migration on human stromal-derived ECM surfaces. **a** Brightfield and immunofluorescent images of PC3 cells transfected with control shRNA vector containing a targeting Endo180 oligonucleotide sequence (PC3-shEndo180); *scale bar* = 100 μm. **b**, **c** Immunoblot analysis (A5/158 mAb) (**b**) and corresponding denistometric analysis (**c**) confirming decreased Endo180 expression in PC3-shEndo180 cells compared to control PC3 cells transfected with a vector containing a non-targeting (scrambled) Endo180 oligonucleotide sequence (PC3-shSCN) (α-tubulin = loading control). **d** Stills extracted from Videos 13–16 in Online Resources 13–16 of PC3-shSCN and PC3-shEndo180 cells migrating on native ECM generated by human HCA2 dermal fibroblasts (fibroblast ECM) and primary human trabecular bone osteoblasts (osteoblast ECM); *scale bar* = 50 μm. **e** The rounded:bipolar ratios of PC3-shSCN and PC3-shEndo180 cells migrating on fibroblast ECM and osteoblast ECM. **f** Migration velocities (μm/h) of PC3-shSCN and PC3-shEndo180 cells migrating for 24 h on human fibroblast-derived ECM and human osteoblast-derived ECM; significant decreases in the velocity of PC3-shEndo180 cells compared to pc3-shSCN cells are indicated (^+^p < 0.01). **g**, **f** The adhesion levels at 1 h (**g**) and proliferation rates at 48 h (**h**) of PC3-shSCN and PC3-shEndo180 cells are shown; significant increases (*p < 0.05) and decreases (^+^p < 0.05) compared to plastic are indicated. **e**–**h** Average ± s.d. values for three individual experiments, within which each experimental condition was tested in quadruplicate, are shown

To test the hypothesis that Endo180 contributes to the plasticity of prostate cancer cell migration, PC3 cells were transfected with a scrambled control shRNA vector (PC3-shSCN) or targeted Endo180 shRNA vector (PC3-shEndo180) (Fig. 5a), which contained a previously validated siRNA oligonucleotide sequence [17, 19, 23, 24, 26, 27, 30] and was highly effective at silencing Endo180 (Fig. 5b, c). PC3-shSCN cells displayed a predominantly bipolar migratory mode on fibroblast-derived ECM and a predominantly rounded migratory mode on osteoblast-derived ECM, as seen in time-lapse videos (Online Resources 13 and 14) and corresponding frames (Fig. 5d, e). PC3-shEndo180 cells displayed a significant transition towards a bipolar morphology on both fibroblast-derived ECM and osteoblast-derived ECM, as seen in videos (Online Resources 16 and 17) and corresponding frames (Fig. 5d, e). Moreover, the rounded-to-bipolar transition that resulted from Endo180 silencing was accompanied by a dramatic reduction in cell velocity (Fig. 5f) associated with defective cell detachment from fibroblast-derived ECM and osteoblast-derived ECM, as seen in videos (Online Resources 15 and 16). The adhesion (Fig. 5g) and proliferation (Fig. 5h) rates of PC3-shSCN and PC3-shEndo180 cells on all substrata were similar to those for parental PC3 cells (Fig. 3b, c). These data confirm that Endo180 promotes rounded prostate cancer cell migration on human stromal-cell derived ECM surfaces.

### Endo180 cooperates with fibroblast-derived LOX to promote metastatic prostate cancer cell migration

**Fig. 6:**
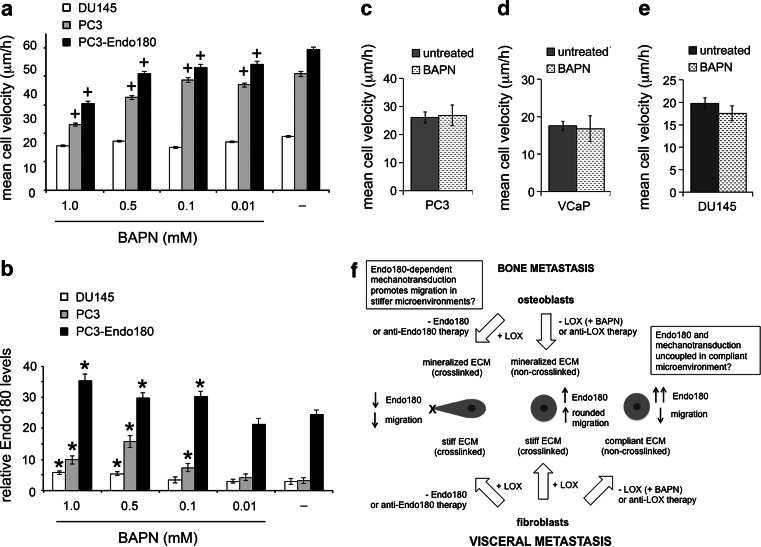
Endo180 requires LOX-dependent ECM crosslinking of human fibroblast-derived ECM surfaces to promote metastatic prostate cancer cell migration. **a** Migration velocities (μm/h) of DU145, PC3 and PC3-Endo180 cells for a duration of 24 h on ECM derived from human HCA2 dermal fibroblasts in the absence (−) or presence of the LOX inhibitor BAPN (0.01–1 mM); significant increases (*p < 0.05) and decreases (^+^p < 0.05) compared to cells migrating on control ECM generated by fibroblasts in the absence of BAPN (−) are indicated. **b** Relative levels of Endo180 expression (integrated fluorescent intensity; A5/158 mAb immunostaining) in DU145, PC3 and PC3-Endo180 cells cultured for 24 h on ECM derived from human HCA2 dermal fibroblasts in the absence (−) or presence of BAPN (0.01–1 mM); significant increases compared to corresponding each corresponding cell line cultured on control fibroblast ECM (−) are indicated (*p < 0.05). **c**–**e** Migration velocities (μm/h) of PC3 (**c**), VCaP (**d**) and DU145 (**e**) cells migrating for 24 h on ECM derived from primary human trabecular bone osteoblasts in the absence (−) or presence of the LOX inhibitor BAPN (1 mM); no significant differences were observed. **a**–**e** Average ± s.d. values for three individual experiments, within which each experimental condition was tested in quadruplicate, are shown. **f** Schematic diagram that summarizes the findings of this study and their potential therapeutic implications in the context of bone and visceral metastasis. Metastatic prostate cancer cells adopt a rounded mode of cell migration on fibroblast-derived and osteoblast-derived ECM that is associated with increased Endo180 expression. Silencing Endo180 (-Endo180) prevents the translocation of prostate cancer cells on fibroblast-derived ECM and osteoblast-derived ECM due to a detachment defect and the potential loss of Endo180-dependent mechanotransduction. Inhibition of fibroblast-derived LOX activity (-LOX) uncouples Endo180 from its pro-migratory capacity, possibly via loss of its capacity for mechanotransduction in the more compliant non-crosslinked ECM. In contrast the inhibition of osteoblast-derived LOX does not affect prostate cancer cell migration, potentially due to the non-crosslinked mineralized ECM being stiff enough to promote Endo180-dependent mechanotransduction and migration. This model supports the use of anti-LOX therapy for metastasis in visceral tissue and anti-Endo180 therapy for metastasis in bone

Given that prostate epithelial cell-associated Endo180 cooperates with AGE-mediated crosslinking of the basement membrane to promote invasiveness [18], we tested the hypothesis that Endo180-dependent metastatic prostate cancer cell migration is promoted by LOX-dependent crosslinking in the stromal ECM associated with bone and visceral tissue. The migratory velocity of parental PC3 and PC3-Endo180 cells, but not DU145 cells, was decreased on ECM generated by confluent monolayers of human HCA2 fibroblasts treated with the LOX inhibitor BAPN (0.01–1.0 mM) when compared to their migratory velocity on ECM generated by untreated HCA2 fibroblasts (Fig. 6a). The decrease in PC3 and PC3-Endo180 cell migration on ECM generated by HCA2 fibroblasts treated with BAPN was correlated with an increase in Endo180 expression (Fig. 6b). However, no difference in the migratory velocity of PC3 cells (Fig. 6c), VCaP cells (Fig. 6d) and DU145 cells (Fig. 6e) was observed on ECM generated by untreated primary human trabecular bone-derived osteoblasts compared to osteoblasts treated with 1.0 mM BAPN (Fig. 6c). These data suggest that metastatic prostate cancer cell migration involves cooperation between tumor-associated Endo180 and LOX-dependent crosslinking in human fibroblast-derived ECM but not osteoblast-derived ECM.

## Discussion

In this paper we explored the role of two emerging anti-metastatic targets involved in collagen matrix homeostasis, Endo180 and LOX, in directing the plasticity of metastatic prostate cancer cells on human ECM surfaces. The in vitro model developed for this purpose involved the introduction of PC3 and VCaP cells originating from metastatic bone lesions [40, 41] and DU145 cells originating from a metastatic lesion in the brain [42] onto human osteoblast-derived and fibroblast-derived ECM. VCaP cells adopted a singular and rounded mode of cell migration, and DU145 cells a singular and bipolar mode of cell migration, on fibroblast-derived ECM. This contrasted with their grouped migratory mode under standard culture conditions on tissue culture plastic or a low concentration of non-polymerized reconstituted type I collagen. Moreover, all three prostate cancer cell lines adopted a singular and rounded mode of cell migration on osteoblast-derived ECM (Table 2). These changes in prostate cancer cell migration on human stromal cell-derived ECM closely recapitulate the ‘amoeboid-like’ mode of migration of tumor cells observed within 3-D lattices formed by high concentrations of polymerized reconstituted type I collagen [11, 43, 44]. These findings suggest that the therapeutic strategies uncovered in these earlier studies may be useful in blocking rounded tumor cell migration and diseemination in MBD and other types of bone cancer.

**Table 2:** Migratory mode, velocity, adhesion and proliferation rates of human prostate cancer cell lines on human fibroblast and osteoblast-derived matrices

Substratum	PC3 cells	VCaP cells	DU145 cells
Tissue culture plastic	Singular, bipolar	Grouped, epithelioid	Grouped, epithelioid
Commercial type I collagen	Singular, Bipolar ⇑ Velocity ⇔ Adhesion ⇔ Proliferation	Singular, bipolar ⇔ Velocity ⇔ Adhesion ⇔ Proliferation	Grouped, epithelioid ⇔ Velocity ⇓ Adhesion ⇓ Proliferation
Fibroblast ECM	Singular, mixed ⇔ Velocity ⇓ Adhesion ⇔ Proliferation	Singular, rounded ⇓ Velocity ⇔ Adhesion ⇓ Proliferation	singular, bipolar ⇑ Velocity ⇔ Adhesion ⇓ Proliferation
Osteoblast ECM	Singular, rounded ⇑ Velocity ⇓ Adhesion ⇓ Proliferation	Singular, rounded ⇓ Velocity ⇔ Adhesion ⇓ Proliferation	Singular, rounded ⇑ Velocity ⇓ Adhesion ⇓ Proliferation

Definition of migratory modes: grouped (>80 % of cells migrate as part of an epithelioid cluster); bipolar (rounded/bipolar ratio <1.0); rounded (rounded/bipolar ratio >1.0); mixed (rounded/bipolar ratio = 1.0). All changes shown are in comparison to plastic

*ECM* extracellular matrix

The intracellular mechanisms of rounded tumor cell migration delineated so far have been centered upon the suppressor and activator signals that regulate RhoA-ROCK and myosin light chain-2 (MLC2)-dependent actinomyosin-based contractility, cytoskeletal remodeling and dynamic cell adhesion events. For example, it has been demonstrated that rounded cell movement can be reversed by Smurf-1, a E3 ubiquitin ligase that targets RhoA for degradation, and PDK1, which antagonizes the RhoE-dependent activation of ROCK [45, 46]. Rounded cell migration is also driven by aberrant activation of RhoA following loss of p53 and p27, the suppression of Rac1 and SOX2, or the expression of EphA2 [43, 47–52]. The interaction between RhoC and FMNL2, Cdc42 and its regulators (DOCK10, RasGRF2) and effectors (N-WASP, PAK2), also promotes rounded cell migration [44, 53–56]; whereas the dephosphorylation of stathmin (a microtubule destabilizing protein), loss of cofilin or depletion of paxillin can block rounded cell migration [57–59].

In this study we have pinpointed the collagen receptor Endo180 as a novel modulator of rounded tumor cell migration in the context of the bone (osteoblast-derived ECM) and visceral tissue (fibroblast-derived ECM) microenvironments. This novel pro-migratory mechanism is consolidated by the upregulation of Endo180 expression by up to ~20-fold in PC3 cells, ~9-fold in VCaP cells and ~7-fold in DU145 cells on osteoblast-derived ECM compared to control substrata. The possible intracellular cues that can direct this Endo180-associated tumor cell plasticity include Cdc42 and Rac1 and the Rho-ROCK-MLC2 pathway, which are activated by the spatiotemporal localization of the Endo180 receptor to the plasma membrane or constitutively recycling endosomes [18, 19, 26]. Interestingly two key Endo180 interaction partners, CD147 and urokinase-type plasminogen activator receptor (uPAR) [17, 26], have been identified as regulators of rounded cell migration [60, 61]. CD147-annexin II complex acts as a molecular switch that directs rounded-to-bipolar transitions during cell migration. It is feasible that Endo180-CD147 complex [17] also plays a modulatory role in tumor cell plasticity on human stromal cell-derived ECM. In this respect we hypothesize that Endo180-CD147 complex disruption can promote rounded tumor cell migration and Endo180-CD147 complex formation can uncouple Endo180 and the intracellular machinery that drives rounded tumor cell migration. It is also possible that the integrin-dependent actomyosin contractile signals generated at the pseudo-uropod-like structure at the rear of spheroidal cells during rounded cell migration [14] involves the spatiotemporal activation of Rho-ROCK-MLC2-based contractile signals by Endo180-containing endosomes. This prediction is supported by the fact that de-adhesion of the uropod at the rear of MG63 osteosarcoma cells requires the Endo180-Rho-ROCK-MLC2 signalling axis [19]. It will also be interesting to consider if the strong Endo180 clusters in PC3-Endo180 cells on osteoblast-derived ECM contribute to their rounded mode of migration.

The requirement of LOX-dependent ECM crosslinking and stiffness for Endo180-dependent tumor cell migration is aligned with the finding that non-enzymatic crosslinking of basement membrane matrix coupled with Endo180-dependent mechanotransduction triggers epithelial cell invasiveness [18]. When considering the design of Endo180 based anti-metastatic therapies it will be important to fully explore the relative contributions of the two functional C-type lectin domains (CTLDs) in the receptor, CTLD2 and CTLD4, to the migratory behavior of metastatic prostate cancer cells in the context of human ECM lattices that have different levels of stiffness. Our findings indicate that where the ECM is more compliant Endo180 and CD147 form a molecular complex that involves CTLD4 and suppresses epithelial cell invasiveness [17]. This suggests that in compliant tissue it would not be desirable to target CTLD4. On the flipside, blockade of CTLD2-dependent mechanotransduction, which can inhibit the epithelial cell invasiveness induced by non-enzymatic crosslinking and increased stiffness of the basement membrane [18], could be used to prevent rounded tumor cell migration in stiff visceral tissue and bone.

In contrast to the finding that Endo180 is uncoupled from its ability to promote tumor cell migration on compliant (non-crosslinked) fibroblast-derived ECM, no differences were observed in the migration of tumor cells on non-crosslinked versus crosslinked osteoblast-derived ECM. Although our findings suggest that osteoblast-derived LOX does not affect metastatic prostate cancer cell migration, tumor-derived LOX participates in the progression of osteolytic bone metastasis in breast cancer [38]. In the current study we did not consider the cooperative roles of tumor-derived LOX and Endo180 in driving the plasticity of tumor cell movement on human fibroblast-derived and osteoblast-derived ECM surfaces. Consideration of this possibility together with the evaluation of Endo180 and LOX as targets in pre-clinical models of osteolytic bone tumors induced by PC3 and DU145 cells [62–64] and predominantly osteosclerotic tumors induced by VCaP cells [65] will be prioritised in our future work. The finding that LOX-dependent crosslinking of human fibroblast-derived ECM is required to promote tumor cell migration, indicates that anti-Endo180 and/or anti-LOX therapy is a feasible therapeutic option for the treatment of visceral tumors surrounded by a stiffened stroma (Fig. 6f).

The findings of this study provide new insight into the consequences of Endo180 upregulation on prostate tumor cells in contact with osteoblasts [4], positive Endo180 immunostaining of tumor cell foci in metastatic bone lesions [4] and raised levels of soluble Endo180 in the serum of patients with osseous and/or visceral metastases [31]. The heterotypic interaction of osteoblasts with prostate cancer cells was previously shown to suppress Endo180 expression in the osteoblasts resulting in decreased mineralized collagen production [4]. Here we have demonstrated that osteoblast-derived ECM increases Endo180 expression in tumor cells to drive their transition to a rounded mode of migration. Therapeutic strategies that can suppress Endo180 function in metastatic disease (Fig. 6f), combined with the development of Endo180-targeted diagnostics, could provide the opportunity to make a major advance in the personalized treatment of men with Endo180-positive prostate cancer who are at risk of, or have progressed towards, the development of Endo180-driven bone metastasis [17, 18].

## Electronic supplementary material

Figure S1 Endo180 is upregulated in metastatic prostate cancer cells cultured on human stromal cell-derived ECM surfaces. Immunoblots show Endo180 expression (A5/158 mAb) in PC3 cells (a) and DU145 cells (c) cultured on rat-tail type I collagen (collagen), osteoblast ECM and fibroblast ECM for 6, 24 and 48 h (GAPDH = loading control). Graphs show relative levels of Endo180 expression in PC3 cells (b) and DU145 cells (d) quantified using densitometry (n = 1)

Online Resource 1—Video shows PC3 cells on plastic; 2 frames/h; 24 h duration; 6 frames/sec

Online Resource 2—Video shows VCaP cells on plastic; 2 frames/h; 24 h duration; 6 frames/sec

Online Resource 3—Video shows DU145 cells on plastic; 2 frames/h; 24 h duration; 6 frames/sec

Online Resource 4—Video shows PC3 cells on collagen; 2 frames/h; 24 h duration; 6 frames/sec

Online Resource 5—Video shows VCaP cells on collagen; 2 frames/h; 24 h duration; 6 frames/sec

Online Resource 6—Video shows DU145 cells on collagen; 2 frames/h; 24 h duration; 6 frames/sec

Online Resource 7—Video shows PC3 cells on fibroblast ECM; 2 frames/h; 24 h duration; 6 frames/sec

Online Resource 8—Video shows VCaP cells on fibroblast ECM; 2 frames/h; 24 h duration; 6 frames/sec

Online Resource 9—Video shows DU145 cells on fibroblast ECM; 2 frames/h; 24 h duration; 6 frames/sec

Online Resource 10—Video shows PC3 cells on osteoblast ECM; 2 frames/h; 24 h duration; 6 frames/sec

Online Resource 11—Video shows VCaP cells on osteoblast ECM; 2 frames/h; 24 h duration; 6 frames/sec

Online Resource 12—Video shows DU145 cells on osteoblast ECM; 2 frames/h; 24 h duration; 6 frames/sec

Online Resource 13—Video shows shSCN-PC3 cells on fibroblast ECM; 2 frames/h; 24 h duration; 6 frames/sec

Online Resource 14—Video shows shSCN PC3 cells on osteoblast ECM; 2 frames/h; 24 h duration; 6 frames/sec

Supplementary material 16 (MOV 2041 kb)

Online Resource 15—Video shows shEndo180 PC3 cells on fibroblast ECM; 2 frames/h; 24 h duration; 6 frames/sec

## Abbreviations

AGE: Advanced glycation end-product
BAPN: Beta-aminopropionitrile
BSA: Bovine serum albumin
CTLD: C-type lectin domain
DOCK10: Dedicator of cytokinesis 10
ECM: Extracellular matrix
EMT: Epithelial to mesenchymal transition
EphA2: Ephrin type-A receptor 2
FBS: Fetal bovine serum
FMNL2: Formin-like 2
GAPDH: Glyceraldehyde 3-phosphate dehydrogenase
GFP: Green fluorescent protein
HRP: Horse radish peroxidase
IFB: Immnofluorescence buffer
LOX: Lysyl oxidase
MBD: Metastatic bone disease
MTT: 3-(4,5-Dimethylthiazol-2-yl)-2,5-diphenyltetrazolium bromide
MLC2: Myosin light chain-2
N-WASP: Neuronal Wiskott–Aldrich syndrome protein
PAK2: p21 protein (Cdc42/Rac)-activated kinase 2
PBS: Phosphate buffered saline
PDK1: Pyruvate dehydrogenase kinase, isozyme 1
RasGRF2: Ras protein-specific guanine nucleotide-releasing factor 2
ROCK: Rho associated protein kinase
shSCN: shEndo180 scrambled control
SOX2: SRY (sex determining region Y)-box 2
TGFβ_1_: Transforming growth factor-beta-1
TGFβ_1_R: Transforming growth factor-beta-1 receptor
uPARAP: Urokinase plasminogen activator receptor associated protein
