# Supplementary figures and images for: Tumor-associated Endo180 requires stromal-derived LOX to promote metastatic prostate cancer cell migration on human ECM surfaces
Source: Clin Exp Metastasis. 2015 Nov 13;33:151–65. doi: 10.1007/s10585-015-9765-7 (PMC4761374; doi:10.1007/s10585-015-9765-7)

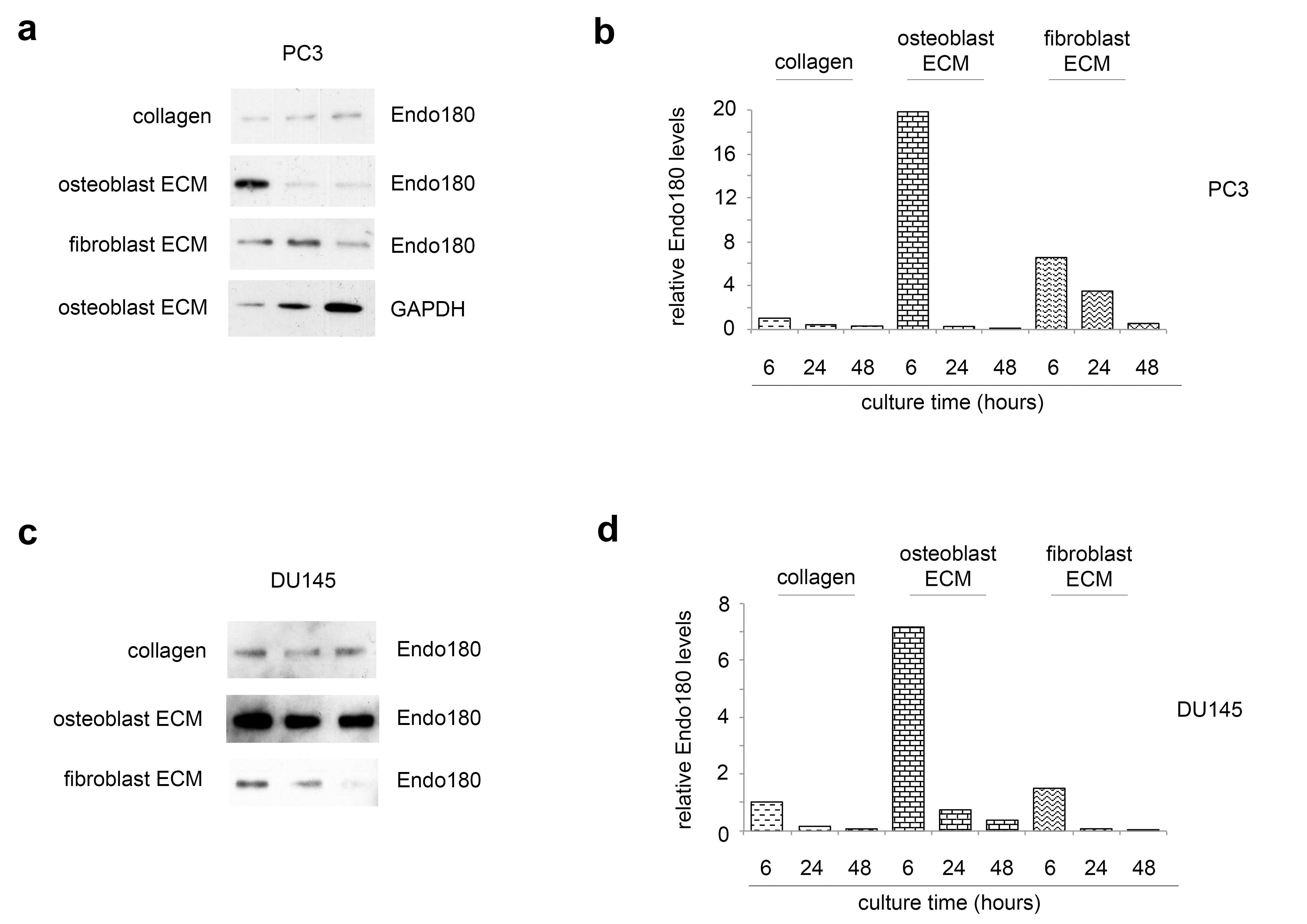

Supplement: Supplementary file 1 — Figure S1 Endo180 is upregulated in metastatic prostate cancer cells cultured on human stromal cell-derived ECM surfaces. Immunoblots show Endo180 expression (A5/158 mAb) in PC3 cells (a) and DU145 cells (c) cultured on rat-tail type I collagen (collagen), osteoblast ECM and fibroblast ECM for 6, 24 and 48 h (GAPDH = loading control). Graphs show relative levels of Endo180 expression in PC3 cells (b) and DU145 cells (d) quantified using densitometry (n = 1) [file 10585_2015_9765_MOESM1_ESM.tif]
